# PREVALENCE AND LONGITUDINAL PERSISTENCE OF PAIN IN DEPARTMENT OF VETERANS AFFAIRS (VA) NURSING HOMES

**DOI:** 10.1093/geroni/igae098.2499

**Published:** 2024-12-31

**Authors:** Linda Am, Wildjena Jean Jacques, Shibei Zhao, Emma Quach, Christine Hartmann, Camilla Pimentel

**Affiliations:** Department of Veterans Affairs Bedford, Bedford, Massachusetts, United States; University of Massachusetts Lowell, Lowell, Massachusetts, United States; Center for Healthcare Organization and Implementation Research, Bedford, Massachusetts, United States; VA Bedford Healthcare System, Bedford, Massachusetts, United States; VA Bedford Healthcare System & University of Massachusetts Lowell, Bedford, Massachusetts, United States; VA Bedford Healthcare System, Bedford, Massachusetts, United States

## Abstract

Pain is highly prevalent in U.S. nursing homes, with estimates of 22%-85% of residents experiencing any pain (Cole et al., 2022). Current studies about pain in Department of Veterans Affairs (VA) nursing homes offer information about a single point in time. Identifying whether pain improves or worsens is critical but difficult. We evaluated prevalence and longitudinal patterns of pain among residents of VA’s 134 nursing homes. We used individual-level VA data from the Minimum Data Set 3.0 from October 1, 2018 to September 30, 2019 (N=40,954). We limited analyses to residents with long length of stay (>100 days); admission, quarterly, or annual clinical assessments (A0310=1,2,3); not comatose (J0200=1); and recent self-reported/observed pain (J0300=1). We randomly selected an “index” assessment for each resident and evaluated pain in ≥2 subsequent, consecutive assessments. We defined “persistent pain” as pain consistent across assessments and “intermittent pain” as pain documented in only one assessment. Our analytic sample was 1,824 long-stay VA nursing home residents. Pain was documented among 900 (49.3%) residents, among whom 509 (56.6%) had persistent pain. Among residents with intermittent pain (N=391; 21.4%), 298 (76.2%) reported worsening pain, and 93 (23.8%) reported improvement in pain. This study demonstrates high prevalence of any pain and persistent pain in VA nursing homes, with experiences of pain potentially worsening over the course of a nursing home stay. Results are consistent with studies in non-VA nursing home settings, underscoring the need to address pain more effectively in all long-term care settings.

